# Neglected, Drug-Induced Platinum Accumulation Causes Immune Toxicity

**DOI:** 10.3389/fphar.2020.01166

**Published:** 2020-08-12

**Authors:** Yuling Zhang, Jieting Zheng, Yi Jiang, Xuchun Huang, Ling Fang

**Affiliations:** ^1^Laboratory of Environmental Medicine and Developmental Toxicology, Shantou University Medical College, Shantou, China; ^2^Pharmacy Department, Cancer Hospital of Shantou University Medical College, Shantou, China; ^3^Digestive Medical Oncology, Cancer Hospital of Shantou University Medical College, Shantou, China; ^4^Department of Clinical Laboratory Medicine, Cancer Hospital of Shantou University Medical College, Shantou, China; ^5^Pharmacy Intravenous Admixture Service, Cancer Hospital of Shantou University Medical College, Shantou, China

**Keywords:** platinum, accumulation, immune, toxicity, drug-induced

## Abstract

Previous studies only focused on different adverse reactions caused by various platinum drugs, but not on common immunotoxicity caused by the accumulation of elemental platinum. Here, we determined the serum platinum concentrations of cancer patients after a metabolism period of platinum drug chemotherapy, in addition to hematological indices and subsequent immune-related adverse reactions, then analyzed the correlations between platinum accumulation, immune cell levels, and immune-toxicity. We chose the day before the next round of chemotherapy as the specified time point for blood sampling. Samples were collected at five time points, separately in oxaliplatin and cisplatin groups. The median serum platinum concentrations in all patients was 294.8 (205.6, 440.3) μg/L, and was approximately two-fold greater in the cisplatin group than in the oxaliplatin group (429.3 vs. 211.7 μg/L). The platinum level of both groups peaked at the third time point, with the average of females being higher than males (383.9 vs. 266.5 μg/L), and was positively correlated with leukocyte and platelet counts, but negatively correlated with erythrocyte counts and concentration of hemoglobin. The risks of anemia and adverse reactions were individually increased by 0.002- and 0.007-fold for every μg/L increase of platinum concentration. To our knowledge, this is the first study on the relationship between platinum accumulation, immune cell levels and toxicity, showing that drug-induced platinum accumulation may interfere with immune cells and thus increase the risk of toxicity.

## Introduction

There are 18.1 million new cancer cases and 9.6 million cancer deaths every year, as estimated by WHO (Bray et al., 2018). Platinum is the most widely used antitumor drug with a long history of drug development, including the first-generation cisplatin, second-generation carboplatin and nedaplatin, third-generation oxaliplatin and fourth-generation lobaplatin. Although more and more alternative strategies, such as derived nanoparticles, have been produced and implemented (Garofalo et al., 2019), platinum-based drugs still play a dominant role in clinical therapy, including basic, combination therapy and even radiotherapy (Li et al., 2019). It has been reported that platinum antitumor drugs can cause adverse reactions related to neuro-, nephritic, hematic and immune toxicity (Yamauchi et al., 2015; Chopra et al., 2016), with immunotoxicity, including allergy, pruritus, diarrhea, nausea, and emesis, being the most common adverse reaction in our previous investigation, and may be due to the effects of immune system by killing cancer cells (Manohar and Leung, 2018). Prior research has demonstrated inhibition of immune function by chemotherapeutic drugs, but some researchers have shown immune enhancement by platinum drugs (Zitvogel et al., 2008; Bezu et al., 2015; Emens and Middleton, 2015). No matter whether inhibiting or enhancing the immune system, most of the research only focuses on the adverse reactions from one kind of platinum drug, but not the common toxicity from platinum drugs in general, and never consider whether the adverse reactions are due to platinum accumulation. Moreover, some patients display a poorer therapeutic effect after several courses of chemotherapy with the same dosage of platinum drugs, which also may relate to platinum accumulation. Reports indicate that the platinum level is 100–1,000 times higher in cancer patients 20 years after platinum chemotherapy than people without platinum therapy (Chovanec et al., 2017), and type I allergic reactions mediated by IgE in workers may be caused by platinum intake through breathing (Linde et al., 2017). Therefore, we believe that the adverse reactions related to platinum drugs may be due to accumulation of elemental platinum, especially for patients undergoing more courses of therapy, even though the original drug had been completely metabolized. There have been few studies on immune interference or its mechanisms by platinum, although studies of other heavy metals, such as lead and mercury, have appeared. For instance, lead can reduce the CD4^+^ subsets of T cells and the CD4/CD8 ratio, and influence expression of cytokines by Th1, Th2, and T cells. Chronic lead nitrate exposure results in decreased rat erythrocytes, hemoglobin, lymphocytes, and monocytes, and mercury exposure results in release of ROS, which inhibits IL-2-dependent signal transduction and reduces proliferation and survival of T cells (Biswas et al., 2008; Mishra, 2009; Sharma et al., 2010; Jorissen et al., 2013). Concerning erythrocytes mediating immune regulation, the concentration of blood lead negatively correlates with CD44 and CD58 adhesion molecule expression (Huo et al., 2019). Lead also has been reported to correlate with anemia (Hsieh et al., 2017), and it has been suggested that cisplatin, as well as other metals, may participate in oxidative stress, causing inflammation by cytokines (Manohar and Leung, 2018), and finally interfering with immune function. As another heavy metal, platinum could perhaps also interfere with the immune system. We therefore explored the change of related hemocyte levels, as well as adverse reactions, in response to platinum accumulation after complete metabolism of the drug, to establish a mechanism of platinum immune response toxicity and offer insight on immunological mechanisms of platinum toxicity.

## Methods and Materials

### Sample Collection

Cases of cancer patients with platinum drug chemotherapy were randomly recruited after diagnosis by a doctor according to the NCCN (National Comprehensive Cancer Network) in the Cancer Hospital of Shantou University Medical College. All patients were eligible for enrollment and exclusion conditions before medication shown in **Table 1**.

**Table 1:** Recruitment criteria.

Enrollment criteria	Exclusion criteria
Required platinum drug chemotherapy after diagnosis	Metallurgical industry employee Metastatic tumor
Estimated lifetime > 6 months	Pregnant or lactating
Hematological index within the normal range	Prior medication involving platinum or other chemotherapy
Provided informed consent to this research	Accompanied by inflammation

Oxaliplatin has a half-life of 46 h, whereas cisplatin has 72 h. In theory, both drugs should be cleared completely after 5.5 times the half-life, which would be 10.5 days for oxaliplatin and 16.5 days for cisplatin. The time interval of chemotherapy was set at three weeks between rounds, according to drug elimination and the clinical requirements of the NCCN. We chose the day before the next round of chemotherapy as the specified time point for blood sampling and collected samples at five time points for the corresponding courses. Finally, we obtained 187 cases after eliminating invalid samples, 89 for oxaliplatin and 97 for cisplatin, from January to December in 2018. The dosage of oxaliplatin and cisplatin was set by the oncologist according to body surface area and the NCCN, as shown in **Table 3**.

Whole blood was extracted by elbow venous in all cases. A total of 1 ml was extracted in a heparin anticoagulant tube for hematological index analysis, and 3 ml was centrifuged (1,200 g, 3 min) to obtain serum for platinum measurement. Adverse reactions were simultaneously recorded. All protocols in this investigation were approved by the Human Ethics Committee of the Cancer Hospital of Shantou University Medical College, China (2015030907). All patients signed informed consent.

### Hematological Index Analysis

Hematological indices were measured with an automatic blood analyzer (Beckman-LH780, USA) immediately after sample collection by the methods on impedance measurement and cyanated methemoglobin colorimetric procedure.

### Measurement of Serum Platinum

A total of 100 μl serum sample and 900 μl 0.5% nitric acid (65%, guarantee) were mixed by vortexing and quantified by graphite furnace atomic absorption spectrophotometry (Jena ZEEnit 650, Germany). Parameters for the AAS-650 were a 265.9 nm wavelength, 0.2 nm slit width, 8 mA Pt-lamp current and the temperature program was shown in **Table 2**. The limit of detection (LOD) was 31.16 μg/L and accuracy of this method was verified by recoveries between 91.73 and 98.31% from spiked serum samples.

**Table 2:** Temperature program for the AAS-650.

	Temperature/°C	Heating rate/°C/s	Retention time/s
Drying 1	90	5	20
Drying 2	105	3	20
Drying 3	110	2	30
Pyrolysis	1150	250	20
Atomization	2700	700	5
Clean out	2800	500	4

### Adverse Reaction Record at Each Corresponding Time Point

Adverse reactions were assessed and recorded by a clinical doctor at each time point, based on The Common Terminology Criteria for Adverse Events (CTCAE, 5.0). We recorded the adverse reactions, including diarrhea, nausea, emesis and allergy, and classified them into five grades (from 0-no symptoms to 4-severe life-threatening).

### Statistical Analysis

Nonparametric analysis was performed, using the Kolmogorov-Smirnov test, for frequency distribution; data with skewed distributions was represented by median and 25^th^–75^th^ percentile, the comparison of rates was performed by the chi-square test, skewed data was compared by non-parametric testing (Mann Whitney U test), and correlation analysis was performed by Spearman rank correlation analysis. The concentration-response relationship was analyzed by binary logistic regression. All analyses were performed with SPSS 22.0 (IBM Corporation, USA) and GraphPad Prism 7.0 (GraphPad, CA) software. A *P*<0.05 was considered as statistically significant in all analyses.

## Results

### General Characteristics of the Study Population

**Figure 1:**
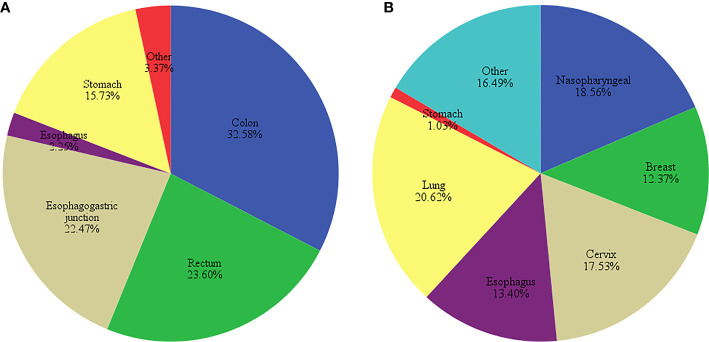
Distribution of cancer types in **(A)** oxaliplatin- and **(B)** cisplatin-treated patients.

In this study, there were 89 patients who were treated with oxaliplatin and 97 with cisplatin, with a higher proportion of female patients and lower average age in the cisplatin-treated group. No significant differences were found in height, weight and BMI between the patients in both groups (*P*>0.05, **Table 3**). Oxaliplatin-treated patients had mostly colon, rectum, and gastroesophageal junction cancers, whereas cisplatin-treated patients had lung, nasopharyngeal and cervix cancers (**Figure 1**).

**Table 3:** General characteristics of the study population.

	Oxaliplatin (n = 89)	Cisplatin (n = 97)	*P*
Gender (males/females)	64/25	51/46	0.007
Height (mean ± SD, cm)	164.9 ± 5.9	163.5 ± 6.2	0.103
Weight (mean ± SD, kg)	55.8 ± 6.5	54.2 ± 7.0	0.107
BMI (mean ± SD, kg/m2)	20.4 ± 1.5	20.2 ± 1.5	0.234
Age (mean ± SD, years)	59.4 ± 10.7	55.8 ± 9.7	0.017
Surgery [n (%)]			0.000
1=No	37 (41.6)	66(68.0)	
2=Yes	52 (58.4)	31(32.0)	
Dose (mg/m^2)	85	65	/

### Platinum Accumulation

#### Serum Platinum of Patients With Different Drugs

**Figure 2:**
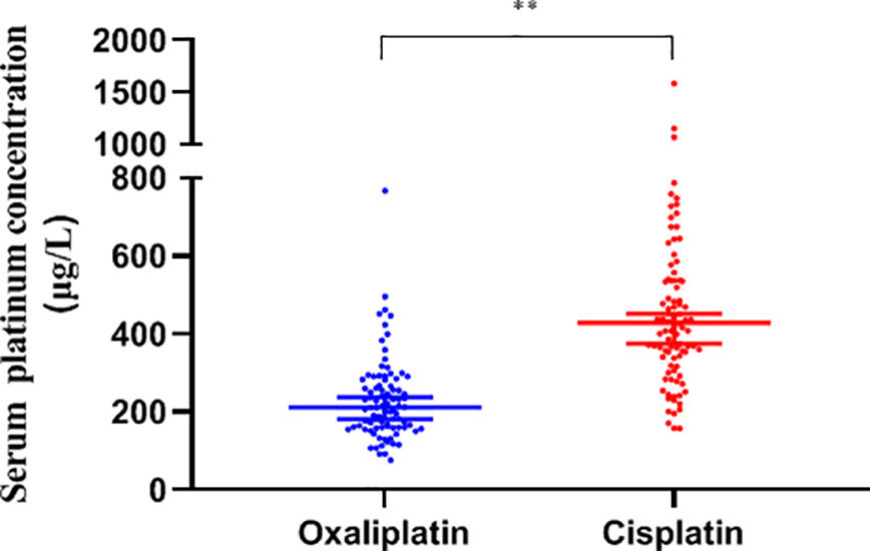
Median serum platinum concentration in each patient group (μg/L). **: *P*<0.01.

The serum platinum concentration of the two groups showed a non-normal distribution and was analyzed by the median. The concentration of platinum displayed a high degree of dispersion with a median of 294.8 (205.6, 440.3) μg/L in all subjects, with the cisplatin group being significantly higher than oxaliplatin, 429.3 (340.3, 539.5) vs. 211.7 (160.9, 275.5) μg/L, (*P*<0.01, **Figure 2**).

#### Serum Platinum at Different Time Points

After analysis of the platinum concentration at each of the five time points, we found significantly different concentrations by the Kolmogorov-Smirnov test between each time point, with peak values obtained at the third point for both oxaliplatin and cisplatin treatments (*P*<0.05, **Table 4**).

**Table 4:** Median serum platinum concentration at each time point.

	1st point	2nd point	3rd point	4th point	5th point	*P* for K-S test
Oxaliplatin	164.1	216.3	251.4	216.1	201.6	0.049
Cisplatin	305.8	476.9	514.8	439.0	417.9	0.001

#### Serum Platinum in Different Genders

We grouped the serum platinum data by gender and found the median level was significantly higher in females than males, with median values being 383.9 (240.0, 496.7) vs. 266.5 (181.9,400.8) μg/L (*P <*0.05) for females and males, respectively.

### Correlations Between Serum Platinum and Hematological Indices

#### Hematological Indices in Two Groups

**Figure 3:**
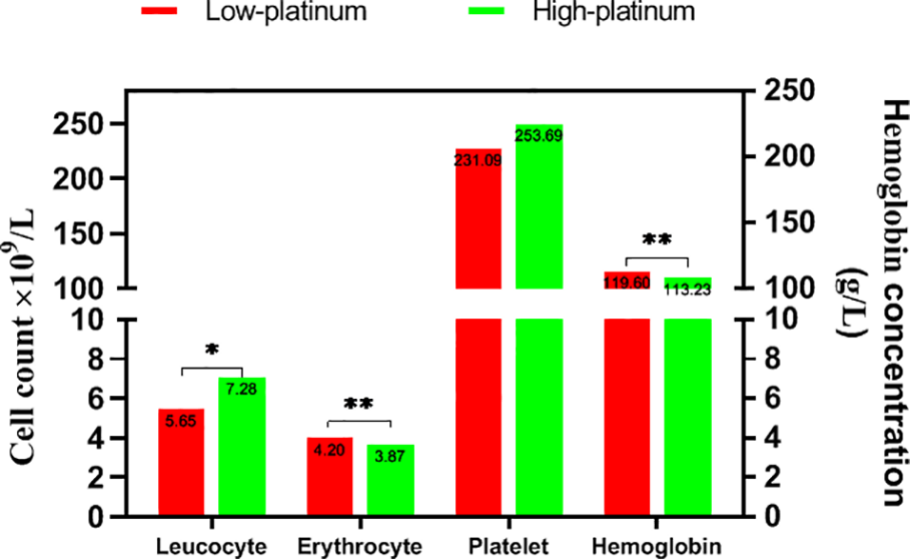
Hematological indices in two groups. **: *P*<0.01; *: *P*<0.05.

We divided the cases into low- and high-platinum groups by the mean of the natural logarithm of platinum concentration, compared the hematological index in the two groups (**Figure 3**). The count of leucocyte was lower in the low platinum group than that in the high platinum group (*P*<0.05). On the contrary, erythrocyte count and hemoglobin took higher levels in the low platinum group (*P*<0.01). There is no significant difference on platelet between the two groups.

#### Correlations Between Serum Platinum and Hematological Indices

**Figure 4:**
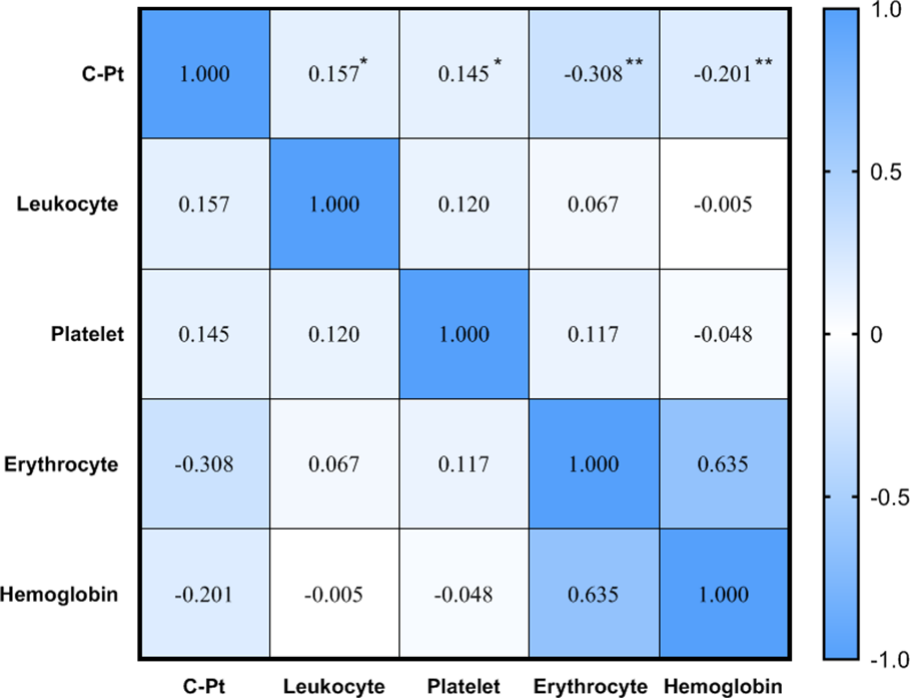
Correlation between serum platinum and hematological indices.**P<0.01; *P<0.05.

Spearman correlation analysis showed a positive correlation between platinum level and leukocyte and platelet counts, but negative correlations between platinum level and erythrocyte counts and hemoglobin (*P*<0.05, **Figure 4**). The current definition of anemia is hemoglobin <120 g/L in adult males, or hemoglobin <110 g/L in adult females (non-pregnancy). In this study, the hemoglobin mean of male and female patients was 119.1 ± 16.4 vs.112.4 ± 12.0 g/L, respectively, with the incidence of anemia being 51.3 vs. 39.4% (no significant difference between male and female groups, *P*>0.05). To determine the correlation between anemia and platinum, a binary logistic regression model was used with occurrence of anemia as a dependent variable (1=no, 2=yes) and platinum as an independent variable. The results showed that the risk of anemia increased 0.002-fold for every unit (μg/L) increase of serum platinum concentration (*P*<0.05, **Table 5**).

**Table 5:** Relationship between anemia and platinum.

	Platinum level		
	OR	95%CI	*P*
Anemia	1.002	1.001,1.004	0.009

### Correlations Between Adverse Reactions and Serum Platinum

We recorded the adverse reactions related to immune toxicity of tumor patients and observed almost no serious events (**Table 6**). However, the chi-square test showed that there was a significant difference between males and females, with the latter displaying a higher incidence of more serious cases (50.7% vs.19.1%, *χ^2^* = 20.393, *p*=0.000).

**Table 6:** Cases of adverse reactions related to immune toxicity.

Adverse events	Nausea		Emesis		Diarrhea		Allergy		Amount	
Gender	Male	Female	Male	Female	Male	Female	Male	Female	Male	Female
Grade										
1	6	10	3	4	2	2	3	3	14	19
2	4	8	1	3	1	2	2	3	8	16
3	0	0	0	0	0	1	0	0	0	1
Amount	10	18	4	7	3	5	5	6	22	36

Spearman correlation analysis showed that serum platinum was positively correlated with the incidence and degree of adverse reactions (*r_s_*=0.466, *P*<0.01; *r_s_*=0.490, *P*<0.01). In order to further analyze the concentration-response relationship between serum platinum and adverse reactions, a binary logistic regression model was used with the occurrence of adverse reaction as a dependent variable (1=no, 2=yes), serum platinum concentration as an independent variable, and following adjustment for gender and age. The results showed that the risk of adverse reactions was increased by 0.007 times for every unit (μg/L) increase of serum platinum concentration, after adjustment with gender, age, surgery, cancer types (*P <*0.05, **Table 7**).

**Table 7:** Relationship between adverse reactions and platinum.

Adverse reaction	Platinum level		
	OR	95%CI	*P*
Model 1	1.007	1.005, 1.009	0.000
Model 2	1.007	1.004, 1.009	0.000

Model 1: unadjuted;

Model 2: adjusted with gender, age, surgery, cancer type.

## Discussion

### Platinum Accumulation

In this study, we found accumulation of platinum in cancer patients after rational interval of chemotherapy and was higher in cisplatin- than oxaliplatin-treated patients (429.3 vs. 211.7 μg/L). It is the first time to compare platinum accumulation of these two drugs, although a study on cisplatin has been reported indicating its continued presence 20 years after therapy (Linde et al., 2017). The original levels of platinum in the two groups were consistent, based on calculations of drug structure and dosage. Differences in platinum accumulation may be due to different drug structures, which would have different binding rates of metabolites with glutathione, cysteine, and ABCC protein (Boisdron-Celle et al., 2001; Wang et al., 2017). Hence, we attribute the difference of platinum accumulation, between cisplatin and oxaliplatin, to molecular structural differences. Moreover, we found large individual and gender differences in platinum levels, perhaps related to polymorphisms of the *GSTP1* and *XRCC1* genes, and drug metabolic diversity *in vivo* (Sawers et al., 2014; Gu et al., 2015). Our finding is consistent with prior research showing that the elimination time for platinum is more than 25 times that for the intact platinum-based drug in the rat (Qin et al., 2019). In addition, there was an increasing trend over the first three time points, which reflected the platinum accumulation. A decreasing trend was observed from the fourth time point, which we suspect was due to greater clearance of the original drug because of the development of resistance to platinum drugs in patients. Most reports have attributed the resistance mechanism to gene mutation or methylation (Alkema et al., 2016; Yan et al., 2017), autophagy induction by cisplatin (Grimaldi et al., 2019), and acquisition of resistance to oxaliplatin accompanied by cross-resistance to another metal such as copper (Martinez-Balibrea et al., 2015). As a result, we consider the trend of platinum distribution as a drug resistance phenomenon caused by platinum accumulation.

### Associations Between Immune Toxicity and Platinum Accumulation

Adverse reactions, such as diarrhea and myelosuppression, in chemotherapy of some platinum drugs (for example: cisplatin, oxaliplatin, or carboplatin) result from toxicity to rapidly dividing cells (Bezu et al., 2015), but more and more data has shown interference of immune system function by platinum drugs. For instance, oxaliplatin and cisplatin can increase production of pro-inflammatory cytokines, such as tumor necrosis factor-α (TNF-α), *in vivo* and stimulate the immune response (Manohar and Leung, 2018; Stojanovska et al., 2019).

Heavy metals can interfere with inflammation mediated by leukocytes and their subsets. For instance, cadmium affects the telomere length of leukocytes and immune regulation (Zota et al., 2015), lead and cadmium may change the counts of leukocytes and their subsets (Zhang et al., 2017). In our research, there were almost no serious adverse reactions of patients, and the more frequent and serious cases were exhibited by the female patients. We also found that residual serum platinum was positively correlated with leukocyte and thrombocyte counts, as well as the incidence of adverse reactions, such as diarrhea, nausea, emesis, and allergy after a period sufficient for drug elimination. Regression analysis further showed the increasing risk of platinum toxicity on immune inflammation mediated by leukocytes.

On the other hand, serum platinum may result in anemia, as shown by the negative correlation with erythrocytes and hemoglobin, and regression analysis showed the risk of increasing platinum on anemia, which is similar to other divalent metals due to a competitive inhibition of iron ion (Hsieh et al., 2017; Weinhouse et al., 2017). Erythrocyte counts are traditionally considered an index of anemia, but more and more research has pointed out its role in immunoregulation. Blood lead has been reported to negatively correlate with adhesion molecules (CD44 and CD58) of erythrocytes (Huo et al., 2019), which hints toward a correlation between platinum accumulation, similar to lead, and immune toxicity mediated by erythrocytes.

In all, we conducted a study on the relationship between platinum accumulation, immunocytes and subsequent adverse reactions. The results show significant correlations between all three, as well as differing toxicities in different genders. All results demonstrate that drug-induced platinum accumulation may stimulate the immune system, as is the case with other heavy metals, and further lead to immune toxicity. Influences on adverse reactions and anemia risk mediated by platinum accumulation suggests that quelation therapy and nourishment to enhance immune function for patients should be considered. The mechanism of platinum resistance is still not clear, but our study suggests that it may due to the platinum accumulation resulting from subsequent rounds of chemotherapy. More and intensive study on the mechanism of immune toxicity caused by platinum accumulation should be carried out in the future.

## Data Availability Statement

The raw data supporting the conclusions of this article will be made available by the authors, without undue reservation.

## Ethics Statement

The studies involving human participants were reviewed and approved by the Human Ethics Committee of the Cancer Hospital of Shantou University Medical College, China (2015030907). The patients/participants provided their written informed consent to participate in this study.

## Author Contributions

LF is the corresponding author of this manuscript. He conceptualized, analyzed the platinum-immune toxicity assessment, performed and wrote the statistical analysis described in this manuscript. YZ is the first author of this manuscript. She measured the concentrations of platinum and analyzed the relationships between the platinum and adverse reactions. JZ collected and stored the blood samples, and administered the questionnaires with patients in the hospital. YJ is the physician of medical oncology. He implemented the therapeutic regimen and assessed adverse reactions of patients. XH is the clinical laboratory doctor. He took responsibilities for the hematological index analysis. All authors reviewed the manuscript.

## Funding

This work was supported by the Science and Technology Plan of Shantou (2016-6) and the Youth Research Fund of the Cancer Hospital of Shantou University Medical College.

## Conflict of Interest

The authors declare that the research was conducted in the absence of any commercial or financial relationships that could be construed as a potential conflict of interest.
